# Taking International Law Seriously? Interpretation of the 1998 Belfast/Good Friday Agreement in the Context of Article 2(1) of the Windsor Framework

**DOI:** 10.1093/ojls/gqag006

**Published:** 2026-02-02

**Authors:** Katie A Johnston

**Keywords:** international law, treaty interpretation, Brexit, constitutional interpretation, Northern Ireland

## Abstract

This article analyses the approach of the Northern Ireland courts to interpretation of the 1998 Belfast/Good Friday Agreement in the context of the ‘no diminution of rights’ obligation in article 2(1) of the Windsor Framework. It advances an alternative approach to the interpretation of the 1998 Agreement that is consistent with international law and provides a more robust basis for rights protection. The article sets out the sources of the rules of treaty interpretation in international law and their application in article 2(1) cases, and analyses the nature of the multi-party agreement part of the 1998 Agreement. It is argued that the multi-party agreement is neither a free-standing treaty nor an integral part of a treaty, and the rules of treaty interpretation do not apply to that instrument. The multi-party agreement, including its Rights, Safeguards and Equality of Opportunity part, should nevertheless be interpreted using a ‘generous and purposive’ approach. In accordance with existing precedents in UK constitutional law, the multi-party agreement should be interpreted generously and purposively due to its nature as a constitution for Northern Ireland.

## 1. Introduction

On 31 January 2020, the UK courts acquired a new power to disapply provisions of primary legislation on human rights grounds. Article 2(1) of the EU–UK Withdrawal Agreement’s Protocol on Ireland/Northern Ireland,^1^ now rebranded as the ‘Windsor Framework’,^2^ provides that:

The United Kingdom shall ensure that no diminution of rights, safeguards or equality of opportunity, as set out in that part of the 1998 [Belfast/Good Friday] Agreement entitled Rights, Safeguards and Equality of Opportunity results from its withdrawal from the Union …

The inclusion of this ‘no diminution’ obligation in the I/NI Protocol was politically significant as it allowed the UK government to maintain that EU withdrawal would not be allowed to undermine the 1998 Belfast/Good Friday Agreement, in particular the protection of human rights.^3^ Now, as the first article 2(1) cases come before the courts, the significant legal implications of this provision for UK constitutional practice, and the UK’s position under international law, are becoming apparent.

Article 4(1) and (2) of the Withdrawal Agreement (WA) creates a binding international obligation for the UK to ensure that the provisions of that Agreement (including its I/NI Protocol) ‘shall produce in respect of and in the United Kingdom the same legal effects as those which they produce within the Union and its Member States’^4^ and, to that end, to ‘ensure compliance … including as regards the required powers of its judicial and administrative authorities to disapply inconsistent or incompatible domestic provisions’. The UK sought to implement these obligations through section 7A of the European Union (Withdrawal) Act 2018,^5^ which essentially recreates the disapplication power (and duty) in section 2 of the European Communities Act 1972, as interpreted by the House of Lords in *Factortame (No 2)*.^6^ Article 2(1) I/NI Protocol is thus given effect in UK law and ‘Every enactment … is to be read and has effect subject to’ it.^7^

The full ramifications of this combination of article 2(1) I/NI Protocol, article 4 WA and section 7A became clear on 28 February 2024, when the High Court in Belfast handed down its decision in *Dillon*, a challenge to the controversial Northern Ireland Troubles (Legacy and Reconciliation) Act 2023.^8^ The judgment was the first in which provisions of an Act of Parliament were disapplied under article 2(1).^9^ The claimants argued that certain provisions of the 2023 Act, notably those allowing for immunity from criminal proceedings and the discontinuation of civil actions, breached article 2(1). As summarised by Mr Justice Colton: ‘In short, any provisions of the 2023 Act which are in breach of the [I/NI Protocol] should be disapplied.’^10^

The article 2(1) cases that have come before the courts so far have raised challenging legal questions, including of treaty interpretation, as discussed below. However, the cases have a wider significance for how NI and the UK will be governed in years to come. NI has in effect acquired its own new human rights protection regime. Although limited by the requirement that, for article 2 to be breached, the diminution in enjoyment of rights must result from the removal of an underpinning in EU law,^11^ these protections go far beyond the applicable Human Rights Act-based legal framework in the rest of the UK; most notably, through the existence of the disapplication power. Thus, ‘Whereas Northern Ireland has long been treated as peripheral within UK constitutional discourse, these [article 2(1)] cases establish it as the most significant proving ground for the interaction of a range of constitutional principles post-Brexit’.^12^

Article 2(1) has so far resulted in disapplication of UK primary legislation in cases involving the rights of victims and the rights of migrants. However, the rights that could benefit from this protection could potentially be much broader. The UK Supreme Court is currently considering the scope of protection offered by article 2(1) in *Dillon*,^13^ but other litigation has come before the NI courts concerning the impact of article 2(1) on the rights of transgender persons,^14^ and the NI Human Rights Commission has advised that ‘the right to personal data protection’ would fall within its scope.^15^ Broad interpretations of the protection offered by article 2(1) could conceivably extend beyond those rights explicitly listed in the text of the 1998 Agreement to encompass other rights underpinned by EU law, such as the right to a clean environment, health or employment. This would amplify the divergence between NI and the rest of the UK and, if UK statutes were regularly disapplied in relation to NI, could potentially disrupt how the UK is governed. Moreover, going beyond article 2(1), the I/NI Protocol makes detailed provision for relations between the EU, the UK, and Ireland post-Brexit, meaning the potential for partial disapplication of conflicting UK statutes could extend to a wide range of areas of governance, from trade to consumer protection.

From an international law perspective, if UK courts misinterpret the I/NI Protocol and 1998 Agreement in article 2(1) cases, the UK risks breaching its international obligations under the WA. That is, if the UK fails to disapply legislation that results in a diminution of those rights, properly interpreted, the UK may be in breach of article 2(1) I/NI Protocol and article 4 WA. Even if under UK constitutional law the Supreme Court is viewed as having the last word on how the I/NI Protocol and 1998 Agreement are to be interpreted and section 7A applied, under international law domestic law cannot be invoked to justify a breach of international obligations.^16^ Crucially, the WA contains its own dispute settlement mechanism, ultimately leading to arbitration,^17^ where the issues around the interpretation of article 2(1) and the nature of the 1998 Agreement discussed below would be analysed using public international law principles.^18^

Turning to the question of treaty interpretation, this issue arises in article 2(1) cases in two interrelated ways. First, to determine whether UK law is in breach of article 2(1) I/NI Protocol, that treaty provision must first be interpreted to determine the content of the obligation it creates. Section 7A instructs that courts give effect to the WA in domestic law; accordingly, the courts must seek article 2(1)’s autonomous international meaning.^19^ Therefore, correct application of the statute under UK law requires interpretation of article 2(1) in line with international law.

Second, article 2(1) explicitly refers to ‘rights, safeguards or equality of opportunity, as set out in that part of the 1998 Belfast/Good Friday Agreement entitled Rights, Safeguards and Equality of Opportunity’. That is, article 2(1)’s meaning is derived in large part from a cross-reference, or *renvoi*, to an external instrument: the rights protected by article 2(1) are those set out in the Rights, Safeguards and Equality of Opportunity (RSEO) part of the 1998 Agreement. Thus, running through all the questions above is the puzzle of how the 1998 Agreement itself should be interpreted. This is most clearly relevant in article 2(1) cases because of the explicit *renvoi*, but the I/NI Protocol contains multiple references to the 1998 Agreement, including provisions requiring the UK and/or the EU to act consistently with or take into account its provisions.^20^ Identifying the 1998 Agreement’s nature, and the correct approach to its interpretation, is therefore essential for interpreting the I/NI Protocol.^21^

The interpretation of the ‘rights, safeguards or equality of opportunity, as set out in that part of the 1998 Agreement entitled Rights, Safeguards and Equality of Opportunity’ is itself complicated by the complex structure of the 1998 Agreement. The 1998 Belfast/Good Friday Agreement comprises: (i) the multi-party political agreement reached on 10 April 1998 following negotiations between the UK government, the Irish government and representatives of political parties in NI (MPA); and (ii) the British–Irish Agreement (BIA), a bilateral international treaty concluded by the UK and the Republic of Ireland. The first annex to the MPA contains a draft of the BIA, which was also signed on 10 April 1998.^22^ The BIA, in turn, includes the MPA as an annex to that treaty. Both instruments are thus annexes of the other, emerging simultaneously from the same negotiations and with their texts agreed as part of the same process. The RSEO part is found within the MPA. Accordingly, further questions arise: what is the correct way to identify the content and scope of the rights set out in the MPA? Is the MPA a treaty or something else and, whatever it is, how should it be interpreted?

Finally, the 1998 Agreement is the document on which NI’s current political settlement is founded. As debate in NI increasingly turns to questions of how the institutions created by the 1998 Agreement could be reformed,^23^ and the conduct of any eventual border poll and perhaps (re)unification,^24^ determining the extent to which the 1998 Agreement should be considered an international treaty creating binding obligations under international law, the requirements for its modification or amendment and how it should be interpreted will be crucial for addressing these future challenges.

All this is to say that the narrow, technical questions of treaty interpretation at issue in the article 2(1) cases to date are but the tip of a constitutional and international legal iceberg, and the broader implications of the approach taken in these cases have perhaps not been fully considered by the courts. With one case currently before the Supreme Court and more cases of this nature certain to come before the courts in future, this article analyses the approach of the NI courts to interpretation of the 1998 Agreement and article 2(1), and advances an alternative approach to interpretation of the 1998 Agreement that is consistent with international law and provides a more robust basis for rights protection.

Section 2 outlines the key article 2(1) cases to date and sets out the sources of the rules of treaty interpretation in both treaty law and customary international law and their application in article 2(1) cases. Section 3 considers how the MPA, including the RSEO section at issue in article 2(1) cases, should be interpreted and analyses the nature of the MPA. It is argued that the MPA is neither a free-standing treaty nor an integral part of a treaty, and that the rules of treaty interpretation codified in the 1969 Vienna Convention on the Law of Treaties (VCLT) do not apply to that instrument. The article then analyses the implications of these conclusions for the article 2(1) cases and the place of the 1998 Agreement in UK constitutional adjudication. Section 4 argues that the MPA, including its RSEO part, *should* be interpreted using a ‘generous and purposive’ approach; but not because it is a treaty to which the VCLT applies. Contrary to the position taken by the courts in some article 2(1) cases to date, even if the MPA were correctly characterised as a treaty, this conclusion would be neither necessary nor sufficient to justify a ‘generous and purposive’ interpretation. The alternative approach, that the MPA should be interpreted using a ‘strict textual’ approach, is also unsustainable. Instead, in accordance with existing precedents in UK constitutional law, the MPA should be interpreted generously and purposively due to its nature as a constitution for NI.

## 2. The Article 2(1) Cases to Date and the Role of International Law

As explained at the outset, a proper understanding and application of the international law rules on treaty interpretation is crucial in article 2(1) cases. Identifying with precision the content of the obligations created by article 2(1) I/NI Protocol, which in turn depends on identifying the scope of the ‘rights, safeguards or equality of opportunity, as set out in that part of the 1998 [Belfast/Good Friday] Agreement’, is a question of treaty interpretation which will determine the scope of the domestic court’s power and duty to disapply conflicting primary legislation. In the High Court in *Dillon*, the answer to the question of whether the MPA is properly interpreted as a treaty, and thus subject to the VCLT interpretation rules which the Court viewed as permitting a more purposive interpretation, was treated as determining whether article 2(1) had been breached in this case and thus whether the relevant provisions of the Legacy Act must be disapplied, and was the subject of detailed discussion. However, in the other article 2(1) cases to date, there has been little analysis of either the nature of the MPA or the applicable rules of interpretation in the context of the 1998 Agreement.

### A. The Pre-*Dillon* Jurisprudence

Prior to *Dillon*, few of the article 2(1) cases to date had grappled with the question of how to interpret the 1998 Agreement generally or the MPA, and none analysed in detail the applicability of the VCLT or the nature of the MPA. This is surprising as the Agreement’s legal status has been debated since its conclusion.^25^ In *In re Ní Chuinneagain*, the High Court judge concluded that ‘There is nothing in that [RSEO] portion of the Belfast Agreement which relates to citizenship rights’, without explaining what interpretative approach was being taken to the 1998 Agreement.^26^ The Court of Appeal accepted without discussion the view that both the BIA *and* the MPA are ‘international agreements’, to be construed in accordance with international law.^27^

In *In re SPUC*, a challenge to regulations implementing abortion services in NI, neither the High Court nor the Court of Appeal articulated their approach to interpretation of the MPA in article 2(1) cases. The High Court judgment referred to the context of the 1998 Agreement as a whole: ‘There is nothing in the phrase “everyone in the community” *or in the rest of the 1998 Agreement*, to suggest this phrase was intended to cover foetuses.’^28^ The Court of Appeal concluded briskly that ‘Plainly, the phrase “everyone in the community” … does not include the unborn’.^29^ Both judgments also noted that article 2 of the European Convention on Human Rights, as interpreted by Strasbourg, excludes foetuses. However, there is no discussion of why these elements are taken into account in interpretation or the interpretative approach being applied, nor of the nature of the MPA. In *In re the Illegal Migration Act 2023*, Humphreys J merely observes that in interpreting the MPA ‘I have not found it necessary to engage with the “generous and purposive approach” advocated by the Vienna Convention on the Law of Treaties’.^30^

By contrast, both *Dillon* decisions go into considerably more detail on issues of treaty interpretation, and the nature of the MPA, than any of the other article 2(1) cases to date. In the High Court, ‘A particular bone of contention between the parties was whether the B-GFA was a treaty/international agreement and, therefore, susceptible to the interpretation principles established by the Vienna Convention on the Law of Treaties’. According to the judge, this question is significant ‘because the VCLT may permit the court to engage in a more purposive interpretation exercise as opposed to adopting a strict textual approach’,^31^ leading the judge to conclude that the term ‘civil rights’ in the MPA encompassed the rights of victims.^32^ This conclusion in turn engages the EU Victims Directive, meaning that the rights which have been subject to diminution are rights ‘underpinned by EU law’, and thus protected by article 2(1).^33^ On 20 September 2024, the Northern Ireland Court of Appeal largely upheld the conclusions and reasoning of the trial judge in *Dillon*.^34^

### B. The Sources of International Law in Article 2(1) Cases

The reasoning of both *Dillon* judgments, although more detailed in their engagement with international law, is nevertheless open to criticism. First, the judgments approach the nature of the MPA through the narrow lens of whether it constitutes a treaty and the VCLT rules therefore apply to it, rather than analysing it as a component of a broader peace settlement. Second, it appears that the judges misapplied—or perhaps, did not clearly explain their application of—foundational international law principles. As the leading case on article 2(1), it would be regrettable if these weaknesses in reasoning were to be echoed in subsequent cases.

The High Court, rejecting the arguments of the UK government, found that ‘the VCLT applies to the interpretation exercise to be carried out by the court’.^35^ However, it is worth noting the novelty of this conclusion: the VCLT ‘has not been cited to any significant extent in previous cases concerning the interpretation of the Agreement’.^36^ Indeed, UK constitutional discourse has been criticised for packaging the 1998 Agreement ‘exclusively into a devolution framework’.^37^ The reasons for the judge’s conclusion that the MPA should be characterised as a treaty will be analysed below. However, even setting this conclusion aside for the moment, there are more fundamental difficulties with the judge’s approach to determining whether the 1969 VCLT applies in article 2(1) cases.

Any international law analysis of the obligations created by the 1998 Agreement, and article 2(1) itself, must be based on a sound understanding of the applicable sources of international law. The WA, a treaty between a state (the UK) and an international organisation (the EU), is incapable of falling within the scope of the VCLT, which applies only to treaties between states.^38^ Rather, treaties like the WA could in principle be governed by the Vienna Convention on the Law of Treaties between States and International Organizations or between International Organizations (VCLT-IO).^39^ However, this treaty is not yet in force and, in any case, although the UK has ratified it, the EU has not. The BIA *is* a treaty between states (the UK and Republic of Ireland), but was concluded in 1998, before Ireland acceded to the VCLT in 2006. Since the VCLT is non-retroactive, the BIA therefore falls outside its scope.^40^ Similarly, even if it is a treaty, the MPA, signed on the same day as the BIA in 1998, also falls outside the VCLT’s temporal scope. It is therefore difficult to accept the judge’s conclusion that the VCLT can apply to any part of the interpretation exercise in *Dillon*, and, by extension, in article 2(1) cases generally.

It is true that the general rules of treaty interpretation in article 31 VCLT and article 31 VCLT-IO are identical, and that this rule also applies as a matter of customary international law to treaties that fall within the scope of either convention. Moreover, unlike the treaty rules, the customary international law rule is not temporally limited; it applies to treaties concluded before the VCLT entered into force and to treaties concluded before the parties to the treaty ratified the VCLT.^41^ Thus, one may object that whether the VCLT applies to the WA or the 1998 Agreement *qua* a rule of treaty law or whether interpretation is governed instead by an identical customary rule that a VCLT or VCLT-IO provision codifies makes no practical difference in this context.

Yet, it is important that domestic judges, in developing the courts’ approach to a significant new, politically sensitive, legal framework such as the I/NI Protocol, set out their reasoning transparently and neither make nor appear to be making elementary errors of international law; namely, failing to distinguish between the application of a rule of customary international law and that of a treaty provision which codifies it. Accuracy in how domestic courts apply the sources of international law is all the more important because their decisions may be a source of the *opinio juris* that constitutes customary international law, as well as subsequent practice in the application of treaties by their parties, which may be relevant to their interpretation.

Although, in substance, the particular rule of treaty law at issue—article 31(1) VCLT—indisputably codifies an identical customary international law rule,^42^ meaning the practical importance of the source being applied is diminished in this case, this is not so for other provisions of the VCLT. The question of whether some provisions codify custom, and whether the contours of the customary and treaty rules are identical, is much more vexed. If, in future, a UK court was asked to determine, for example, the conditions under which the WA or the 1998 Agreement could be terminated, and if the (incorrect) precedent holding that the VCLT applies to those agreements rather than the customary international law of treaties is followed, this error could have a material impact on the content of the rule of treaty law applied by the UK court. This is because it is doubtful that the VCLT and customary international law rules on procedures for termination, suspension and invalidity of treaties are identical in all respects.^43^

Finally, from the perspective of UK law, the source of the interpretative rule in treaty or custom may be significant for its treatment by domestic courts. While it is no longer straightforwardly the case that customary international law automatically forms part of the common law,^44^ international law ‘is one of the sources of the common law’.^45^ As Lord Sumption explained, ‘Although the courts are not bound, even in these contexts, to take account of international law, they are entitled to do so if it is appropriate and relevant’.^46^ By contrast, it is well established that a treaty to which the UK is party does not become part of UK law unless it has been incorporated by legislation.^47^ Although ‘there is a strong presumption in favour of interpreting English law (whether common law or statute) in a way which does not place the United Kingdom in breach of an international obligation’, including treaty obligations,^48^ the common law cannot incorporate a treaty by the back door.^49^ Thus, if the relevant rule of treaty interpretation is found in customary international law, not the VCLT, UK courts may have greater scope to draw on that standard in developing the applicable common law rule for how a treaty should be interpreted.

In *Dillon*, there is evidence the Court of Appeal recognised the complexity of how the sources of law apply in article 2(1) cases. The Court begins with a quotation from Lord Hope recognising that the VCLT reflects customary international law.^50^ However, in subsequent paragraphs, the scope of the VCLT and its interaction with customary international law are addressed only obliquely. The Court makes some laconic observations about the applicability of the VCLT and customary international law, but does not explain clearly which rule of international law is being applied, leaving the reasoning on which the judgment is based opaque. The Court of Appeal observes that ‘The WA is plainly an international treaty to which the VCLT could apply. Both the UK and Ireland are parties to the VCLT.’ The use of the conditional ‘could’ rather than ‘does’ suggests the Court is aware that the VCLT cannot apply to the WA *qua* agreement between a state and an international organisation. Yet, if so, then the fact that the UK and Ireland are parties to the VCLT is irrelevant; and in any case, the WA is a treaty to which Ireland is not a party in its own right. At best, we are left to infer that the Court has considered and applied the international legal principles above.

In the subsequent sentence, the Court of Appeal notes that ‘it is well-recognised that articles contained in this treaty on treaties [the VCLT] represent a codification of the norms of customary international law’.^51^ Yet, as noted above, while many of the rules in the VCLT reflect customary international law, not every article codifies custom. Moreover, the reason why this is important is not explained: the limited material scope of the VCLT means it cannot apply to the WA, and it is instead the customary rule identical to the rule in article 31 VCLT and VCLT-IO that applies to that treaty. Thus, at the least the article 2(1) cases so far demonstrate that there is a need for UK courts to engage better with public international law arguments. Going forward, the courts could easily avoid such confusion through clearer drafting and a more precise account of how the sources of international law apply to these instruments.

## 3. The Nature of the Multi-party Agreement

Clarifying the applicable sources of international law is an important preliminary step for the key question raised by the article 2(1) cases to date and which, besides *Dillon*, has been largely unaddressed: what is the nature of the MPA and how should it be interpreted?

As noted in the introduction, the 1998 Belfast/Good Friday Agreement comprises two instruments: the MPA and the BIA. Such hybrid forms are common in the design of peace agreements, and reflect the need to accommodate the diverse actors involved in negotiations to end a conflict.^52^ Using non-legal instruments in combination with binding international treaties, constitutional changes or domestic legislative measures can create ‘a set of obligations that will best lock a range of state, nonstate, and international actors into a set of future relationships capable of implementing the peace agreement’.^53^ For this reason, ‘relatively few peace agreements fall straightforwardly within the definition of a treaty under the VCLT’;^54^ article 2 defines a treaty as ‘an international agreement concluded between States in written form and governed by international law, whether embodied in a single instrument or in two or more related instruments and whatever its particular designation’.^55^

The decision of the High Court in *Dillon* is the only judgment to carry out a detailed analysis of the nature of the MPA and the correct approach to its interpretation in article 2(1) cases. However, as discussed in section 4, approaching this as a binary question of whether that instrument constitutes a treaty fails to capture the complexity and context of the 1998 Agreement and its role as a constitution for NI within a broader peace settlement. Even assuming, for argument’s sake, that such an approach were correct, there are serious flaws in the reasoning of the High Court by which it reaches the conclusion that the MPA is correctly analysed as a treaty. The MPA, including its RSEO part, *should* receive a generous and purposive interpretation. However, this is not on the basis that the MPA is (part of) a treaty to which the VCLT rules apply, which is difficult to sustain.

In light of the analysis in the previous section, the best approach seems to be to treat Mr Justice Colton’s statements that the VCLT ‘applies’ to the interpretation exercise to be carried out by the Court as meaning rather that the *customary rules codified in the VCLT* apply, and to analyse the arguments on that basis. The judge’s reasons for his conclusion that these rules do apply to the MPA appear to be fourfold, although the arguments overlap to some extent.^56^ Three of these reasons were adopted almost verbatim by the Court of Appeal.^57^ First, the British and Irish governments ‘were involved in, and facilitated’ the talks that led to the conclusion of the MPA. Second, the MPA is annexed to the BIA, a bilateral international treaty. Third, in the BIA, the two governments undertook an obligation to ‘support, and where appropriate implement’ the MPA, and the MPA itself ‘includes obligations on the British and Irish governments’. The only argument of the High Court which the Court of Appeal omits is the final one, that the ‘rights, safeguards and equality of opportunity referred to in the relevant chapter of the B-GFA Agreement are incorporated into Article 2’ I/NI Protocol, which is a treaty provision.

Each of these arguments will be analysed in turn. Yet, with respect, none of them provide convincing support for the position that the rules of treaty interpretation codified in the VCLT apply to the MPA. For this reason, this approach should not be followed in future article 2(1) cases.

### A. State Involvement in Negotiation of the Multi-party Agreement

The first reason given by the judge for his conclusion that the VCLT applies is that ‘The British and Irish governments were involved in, and facilitated, the talks between the parties’.^58^ As acknowledged in the preamble to the BIA, the MPA was an agreement not merely among NI political actors, but among those political actors *and* the two governments.^59^ This raises the possibility that, at least between the two states, the MPA could qualify as a treaty to be interpreted using the rules codified in the VCLT.^60^

However, although it is necessary for an international agreement to be between states to qualify as a treaty for the purposes of the VCLT definition, this is not sufficient. The more important criterion is that an instrument must be ‘governed by international law’. Instruments that create obligations only under domestic law, or merely political undertakings, are not ‘treaties’ for the purpose of the VCLT definition. Such instruments are not invalid and may have legal force under domestic law or other rules of international law.^61^ Indeed, ‘It has long been recognized in international practice that governments may agree on joint statements of policy or intention that do not establish legal obligations’.^62^ However, they will not come within the scope of the rules of treaty interpretation. Thus, mere involvement of the two governments in the talks that led to its conclusion, and even state participation in the final agreement, are not sufficient to conclude that the MPA is an instrument to which the rules of treaty interpretation apply.

### B. The Multi-party Agreement is Annexed to a Treaty

The second reason why the judge concluded that the VCLT applies was that:

The British and Irish governments simultaneously entered into an international agreement welcoming the B-GFA which was annexed to their text. The opening paragraph of the Agreement between the two governments states: ‘Welcoming the strong commitment to the Agreement reached on 10 April 1998, by themselves and other participants in the multi-party talks and set out in Annex 1 to the Agreement (hereinafter “the multi-party agreement “).’ Pursuant to article 31(2) VCLT annexes form part of a treaty for the purposes of interpretation.^63^

In the context of the judgment, as well as argument by the UK government to the contrary, the judge appears to take the view that Annex 1—containing the MPA—is an integral part of the BIA and is thus subject to interpretation as a treaty.^64^

This conclusion is difficult to sustain. Article 31(2) VCLT is a rule about how to interpret treaty provisions to which the VCLT is applicable; it is not a rule intended to provide a general definition of what counts as (an integral part of) a treaty for the purposes of the applicability of the VCLT rules in the first place. That definition is already provided in article 2 VCLT, which says nothing about annexes. Similarly, the phrase ‘for the purpose of interpretation’ in article 31(2) is referring to the interpretation *of the treaty provisions*, not of the annexes, which is at issue in this case. It is therefore a stretch to rely on the phrase ‘the text, including its preamble and annexes’ to reach the more general conclusion that an annex always forms an integral part of the treaty itself, or that the VCLT interpretation rules will necessarily apply to the interpretation *of that annex*. By comparison, article 31(3)(a) VCLT requires that subsequent agreements among treaty parties are taken into account in interpretation. However, it is not suggested, nor would it be reasonable to infer, that these materials thereby form part of the treaty itself, nor that they should be interpreted using those same interpretation rules.

Annexes do not necessarily form integral parts of the treaties to which they are attached. Indeed, ‘Unless there is an express provision that a particular document or documents are integral to the treaty, the presumption is that they are *not*’.^65^ The use of annexes or appendices which can be easily modified and updated without the need for formal treaty amendment is a familiar drafting device in multilateral treaties.^66^ Moreover, the BIA conspicuously does not include the common phrase (found in eg article 19 I/NI Protocol) that its annexes ‘shall form an integral part of’ the treaty. The more specific provision on this point in the BIA (or lack thereof) militates against the judge’s conclusion that the annexes do form an integral part of that treaty based merely on inferences from the generally applicable VCLT provisions that relate to interpretation.

### C. Obligations Imposed on States

The key criterion for determining whether an international agreement is properly qualified as a treaty is whether it is ‘governed by international law’. Villiger writes that ‘the requirement that an agreement is governed by international law embraces the intention of the parties to create international legal obligations rather than non-legally binding statements of policy’.^67^ This seems to be what the judge had in mind with his third reason for concluding that the VCLT applies to the interpretation of the MPA:

In article 2 of the B-GFA, the two governments affirm their ‘solemn commitment to support and, where appropriate, implement the provisions of the multi-party agreement.’ Furthermore, the B-GFA includes obligations on the British and Irish governments. By way of example, the British government agreed to complete incorporation into Northern Ireland law of the ECHR. Similarly, the Irish government agreed to take steps to ‘further strengthen the protection of human rights in its jurisdiction.’^68^

The difficulty here is that the same acronym, B-GFA, is being used to refer to two different parts of the 1998 Agreement. The first sentence is referring to article 2 BIA, an international treaty registered with the UN Secretariat, which unquestionably creates legally binding international obligations for both the UK and Ireland. However, in the remainder of the passage above, ‘B-GFA’ is used to refer to the MPA, whose nature is at issue in *Dillon*: it is paragraph 2 of the RSEO section which provides that ‘The British Government will complete incorporation into Northern Ireland law of the European Convention on Human Rights’, and paragraph 9 which provides that ‘The Irish Government will also take steps to further strengthen the protection of human rights in its jurisdiction’.

Yet, it is this very same section of the MPA—the RSEO section—that the High Court in *Dillon* was being asked to interpret and whose nature it must therefore identify. It is circular reasoning to answer the question of whether the MPA creates international legal obligations for states and must therefore be interpreted as a treaty by pointing to provisions of that same instrument and asserting that they create (presumably international legal) obligations for states. The MPA clearly contains commitments by the two governments, but it is begging the question to say that these are necessarily international legal obligations, rather than political undertakings. There are many agreements reached between states which do not create international legal obligations and thus do not come within the definition of treaty under customary international law and article 2 VCLT.^69^

A more convincing approach would be to argue that the language used in those paragraphs of the RSEO section, and the context in which it was adopted, are such as to evidence an intention by the two governments to create obligations that are binding under international law.^70^ Yet, the language used does not permit a clear inference that these provisions were intended to create international legal obligations. The use of the verb ‘will’, instead of the more usual indicator of legal obligation ‘shall’, as well as the rather vague content of the commitment undertaken by the Irish government (‘take steps to further strengthen’) do not render such an argument impossible, but are not strongly supportive of an intention to create international legal obligations. A more significant counter-argument is based on the context of the MPA: if the two governments understood and intended the MPA *itself* to have created legally binding obligations for the UK and Ireland, then it is unclear why it was also necessary to conclude a bilateral treaty containing what is clearly an international legal obligation to—as the judge points out—‘support and, where appropriate, implement the provisions of the multi-party agreement’.^71^

It is at this point that the MPA’s location within a broader peace agreement becomes relevant to its interpretation. The use of different instruments within a hybrid peace agreement is a drafting strategy employed to address the specific challenges of creating peace settlements, which involve state and non-state actors, and must both set the terms for an immediate cessation of violence and create a framework for longer-term governance. When constructing peace agreements, ‘The difficulty for drafters is to craft obligations that will pin down commitments that are enough to command compliance yet leave some room for the coherent holistic development’.^72^ One means to achieve this is to use multiple instruments with differing legal statuses. To interpret the MPA and the BIA as effectively the same instrument, with the same legal nature and status, erases the choice of the negotiators and drafters as to how the 1998 Agreement is constructed. The origins of the rights provisions in the 1998 Agreement are lengthy and complex, but the choice to include them within the MPA, rather than as a separate declaration or entrenched through statute, appears to have been deliberate.^73^ Thus, again, although not rendering such a conclusion impossible, the context of the MPA should cause an interpreter to question whether it having the same status as, or being part of, the BIA is the most convincing account.

### D. Rights Set Out in the Multi-party Agreement Are Incorporated into Another Treaty

The fourth reason given by the judge for the applicability of the (rules codified in the) VCLT is that:

the court also is engaged in an interpretative exercise of Article 2(1) WF. The rights, safeguards and equality of opportunity referred to in the relevant chapter of the B-GFA Agreement are incorporated into Article 2 which is undoubtedly a treaty provision.^74^

Yet, this is not the question that the judge has been answering with the first three reasons given above: these concerned whether the *MPA* is properly qualified as a treaty, subject to interpretation using the general rule of treaty interpretation codified in article 31 VCLT. There is no doubt that the WA, including its I/NI Protocol, is an international treaty, to be interpreted using the customary rule codified in article 31 VCLT-IO. If it were merely a question of interpreting article 2(1) I/NI Protocol, the analysis would be relatively straightforward. The difficulty arises because of the *renvoi* from article 2(1) to the MPA.

The drafters of the I/NI Protocol did not define the rights to be protected from diminution in the text of article 2(1). Had they done so, identifying the contours of those rights would indeed have been a question of interpreting that provision using the general rule of treaty interpretation. Instead, article 2(1) protects the ‘rights, safeguards or equality of opportunity, *as set out in that part of the 1998 Agreement* entitled Rights, Safeguards and Equality of Opportunity’.^75^ This is a clear example of a *renvoi*: the incorporation of an external legal norm into a treaty. The Court of Appeal appears to recognise as much, stating that: ‘Self-evidently, this article does not articulate each relevant right or safeguard in itself. Rather, it is an obligation of result. It highlights a state obligation regarding established rights which must not suffer diminution post-withdrawal.’^76^

A *renvoi* is a commonly used device in treaty drafting; many treaties contain provisions that refer to other treaties, customary international law or other international instruments to provide some of their content and so ‘provide a clear entry-point for other sources of law’ into the referring treaty.^77^ For example, the VCLT makes six explicit references to the UN Charter. References to externally created standards also feature heavily in the UN Convention on the Law of the Sea.^78^

While the judge is correct that the rights referred to in the relevant chapter of the MPA are incorporated by reference into article 2(1) I/NI Protocol, this does not mean that the external norms referred to somehow become part of or acquire the character of the referring treaty provision. They remain external and must be identified or interpreted in accordance with the relevant rules that govern that type of norm. For example, where a treaty makes a *renvoi* to customary international law, as article 51 of the UN Charter does to the customary right of self-defence, the scope and content of that customary rule must be identified in accordance with the test for the identification of custom, not the rules of treaty interpretation.^79^

As a result, while the interpretation of article 2(1) I/NI Protocol as a whole is of course governed by the general rule of treaty interpretation, identifying the ordinary meaning of the terms of the *renvoi*—the ‘rights, safeguards or equality of opportunity, *as set out in that part of the 1998 Agreement* entitled Rights, Safeguards and Equality of Opportunity’^80^—must be done in accordance with the rules that apply to that external norm. It is this question—what is the MPA and what rules apply to the determination of its content? —that the judge had rightly appeared to be addressing with his first three arguments. It is no answer to this question to observe that the external norm is incorporated into a treaty; that is not determinative of how that norm should be interpreted. It is perfectly possible—indeed, commonplace—for treaties to refer to non-treaty instruments to give content to their provisions. To conclude that the VCLT rules apply to the interpretative exercise in this case because article 2(1) is a treaty provision is to overlook the whole complexity of the issue, and the more convoluted drafting of article 2(1).

The Court of Appeal was therefore correct not to adopt this fourth argument of the judge in its judgment. It would have been preferable for the Court to make clear that this reasoning is not correct. Instead, after reprising the first three arguments above, the Court of Appeal addresses the argument, raised in detail only at the appeal stage, that the I/NI Protocol should be interpreted in line with EU law principles.^81^ This argument does not touch on the incorporation point, and we are left to speculate as to whether the Court omitted to address this point simply for reasons of judicial economy^82^ or because it considered the argument unpersuasive.

For all these reasons, the MPA is not correctly analysed either as an integral part of the BIA or as a free-standing treaty. Not only is such a conclusion unsustainable as a matter of international law, but, looking beyond article 2(1), it is undesirable as a matter of policy: it risks creating legal and practical difficulties for the governance of NI in future. Through the inclusion of an international treaty in the 1998 Agreement, the ‘terms of the [BIA] treaty put the governmental commitments into a legally binding form, so as to underwrite their commitment to comply—not just with the treaty commitments—but with the Agreement as a whole’; the BIA also ‘aimed to underwrite the compliance of a range of other [non-state] parties and actors’.^83^ International treaties are able to perform this ‘underwriting’ function due to both the legally binding nature of treaty obligations, as failure to comply amounts to an internationally wrongful act entailing the responsibility of the wrongdoing state,^84^ and the fact that they are relatively difficult to get out of. Treaty obligations can be terminated or modified, but both processes typically require the consent of the other parties to the treaty, and often must follow a formal process.^85^ While it may be these qualities that make treaty obligations a desirable component of a peace settlement in its early stages as a means to promote compliance and reassure other parties, they may create tensions with the longer term goals of peace agreements, such as the coherent development of the peace settlement over time.^86^

In the 1998 Agreement context, if the MPA were an international treaty, any departure from its text would need to comply with the legal processes for treaty modification or be characterised as a breach. This would impose significant practical, legal and political hurdles to future reform or evolution of the peace settlement, potentially undermining stability in the long term. Moreover, analysing the MPA as a treaty is difficult to reconcile with the departures from its text that have occurred since 1998. If the MPA is a treaty, it is difficult to explain how later political agreements, such as the St Andrews Agreement and the Stormont House Agreement, which altered the Strand One institutions such as the NI Assembly, could have modified its provisions.^87^

For those who maintain that the MPA is an international treaty out of fear that to do otherwise would undermine state compliance with its provisions, such a view is understandable,^88^ but would be to build a house on sand. It is not sustainable in international law to argue that the MPA is a treaty or itself creates binding obligations under international law, and it is consequently unlikely that an arbitral panel made up of international lawyers would be convinced that this is the case. A better analysis is that article 2 BIA obliges the UK and Ireland under international law to ‘support, and where appropriate implement’ the MPA, but that the provisions of the latter do not themselves constitute international obligations. This preserves the benefits of having MPA commitments underwritten by international obligations, while explaining past departures from the text of the MPA and allowing some flexibility for reforms to be made without an onerous process of treaty change.

## 4. Interpreting the Multi-party Agreement: Beyond the VCLT

In interpreting the scope of the rights protected by article 2(1) I/NI Protocol, the MPA should be interpreted generously and purposively by UK courts, but characterising that instrument as a treaty is neither necessary nor sufficient to justify such an approach. Instead, a generous and purposive reading is justified by the MPA’s nature as a *sui generis* document that functions as a constitution for NI. This does not depend on the doubtful conclusion that the MPA is a treaty and thus also avoids setting up problems further down the line for, *inter alia*, reform of the 1998 Agreement.

### A. The Interpretative Approach under Article 31(1) VCLT

The phrase ‘generous and purposive’ in *Dillon* echoes the language used by Lord Bingham in *R (European Roma Rights) v Prague Immigration Officer*, explaining the correct approach to interpretation of the 1951 Refugee Convention:

Lord Lester urged that the Convention should be given a generous and purposive interpretation, bearing in mind its humanitarian objects and purpose clearly stated in the Preamble quoted in full in para 6 above. This is, in my opinion, a correct approach to interpretation of a Convention such as this and it gains support, if support be needed, from article 31(1) of the Vienna Convention on the Law of Treaties which, reflecting principles of customary international law, requires a treaty to be interpreted in the light of its object and purpose.^89^

However, although it has long been the refrain of the UK courts that the VCLT takes a ‘generous and purposive’ approach to interpretation, it is doubtful that this phrase accurately captures the nuances of treaty interpretation under the VCLT rules. Indeed, the danger of such labels is that ‘these become shorthand substitutes for understanding and applying the actual Vienna rules’.^90^

The VCLT was based on draft articles on the law of treaties, developed by the United Nations International Law Commission (ILC) between 1953 and 1966. Draft article 70(2), the forerunner of article 31(1) VCLT, when presented by the ILC Special Rapporteur on the law of treaties, Sir Humphrey Waldock, differed from the final version in giving primacy to a literal meaning. The other elements—context, object and purpose, and subsequent practice and agreement—were ‘relegated to a second rank’.^91^ Over the course of the ILC debates and negotiations at the Vienna conference, this was abandoned in favour of the ‘single combined approach’ of article 31(1) VCLT, in which famously all the elements—ordinary meaning, object and purpose, context and good faith—‘would be thrown into the crucible, and their interaction would give the legally relevant interpretation’.^92^ However, while the general rule of interpretation codified in article 31 VCLT thus not only permits but *requires* an interpreter to consider the object and purpose of a treaty, this is far from a licence to engage in broad teleological interpretation.^93^

In *Libya/Chad*, the International Court of Justice held that ‘Interpretation must be based above all upon the text of the treaty’.^94^ Indeed, the ‘object and purpose’ of a treaty itself has a specific technical meaning in this context—it is typically derived from the text of the preamble and initial general clauses of a treaty. Treaty interpretation should thus be distinguished from contractual and statutory interpretation in some domestic systems, which seeks the actual or presumed intention of the parties or drafters. Treaties, which may have been drafted in a highly politicised context, perhaps with over 100 negotiating parties, each with their own understanding of what the treaty provisions mean and its object and purpose, have been described as ‘a disagreement reduced to writing’.^95^ For this reason, the *travaux préparatoires* of a treaty are given a supplementary role in the interpretative process by article 32 VCLT, and while recourse ‘may’ be had to them in certain situations, it is not required that they be considered at all.^96^ To the extent that a treaty is interpreted in accordance with the intention of its drafters, this is only the intention ‘as reflected by the text of the treaty and the other relevant factors in terms of interpretation’.^97^

Moreover, article 31(1) VCLT requires that *all* the elements in that provision be taken into account in interpretation: ordinary meaning of the terms; context; object and purpose; and good faith.^98^ Even though article 31(1) does not embody a primarily literal or textual approach, the object and purpose of a treaty cannot be used to justify disregarding the other elements.^99^ In this way, treaty interpretation under the Vienna rules may be distinguished from the interpretative approach of the Court of Justice of the European Union in relation to EU law, in which a teleological interpretation is prioritised, and is generally decisive.^100^

Thus, even if the MPA were correctly characterised as a treaty, it does not necessarily follow that it must be given a ‘generous’ interpretation by domestic courts applying article 2(1) in cases that may come before them. While the VCLT interpretation rule may to some extent mandate a ‘more purposive’ approach,^101^ this is only within the limits permitted by a textual approach to interpretation, and it is doubtful whether the overall interpretative approach should be described as particularly ‘generous’. In *Roma Rights*, Lord Bingham goes on to add the explicit caveat that ‘However generous and purposive its approach to interpretation, the court’s task remains one of interpreting the written document to which the contracting states have committed themselves’.^102^ More recently, when interpreting the WA itself, the Court of Appeal in England and Wales has noted that:

When article 31 of the Vienna Convention attaches weight to the ‘object and purpose’ of the instrument this is not an invitation to delve into the minefield of negotiating objectives as a substitute for focusing upon the ‘text as a source for determining the parties’ intentions’.^103^

In any case, in *Roma Rights* Lord Bingham also included the implicit qualification that a generous and purposive approach is (only) appropriate for a convention ‘such as this’, whose object and purpose is humanitarian in nature.^104^ This may reflect the ‘special emphasis’ given to the object and purpose when interpreting human rights treaties using the rule in article 31(1) VCLT, although it still remains doubtful whether this approach is correctly described as ‘generous’.^105^ In any case, this approach should be contrasted with other UK cases interpreting treaties which do not have humanitarian/human rights purposes. In *Al-Malki*, when interpreting the Vienna Convention on Diplomatic Relations, Lord Sumption correctly applies the rule in article 31(1) VCLT and notes the importance of the object and purpose, but the phrase ‘generous and purposive’ is absent.^106^ Thus, even if the MPA is correctly characterised as a treaty, to be interpreted in accordance with the rule in article 31(1) VCLT, it is only if its object and purpose is of a humanitarian or human rights character that, according to the UK courts, the interpretation should be generous and purposive. There is clearly a strong argument that, if it were a treaty, the MPA should be so understood, given that it enumerates the rights at issue in article 2(1) cases. However, this step has not been addressed explicitly in the article 2(1) cases to date.

It could be argued that the 1998 Agreement’s function as a peace agreement justifies departure from the international law rules of treaty interpretation set out above, for example, so as to justify a particularly generous interpretative approach. Scholars have argued that peace agreement practice in recent decades has impacted international law so as to ‘recast its norms’^107^ or that in post-conflict transition ‘the ordinary intuitions and predicates about law simply do not apply’, generating ‘a *sui generis* paradigm of transformative law’.^108^ Yet, there is limited support in practice for the emergence of a *lex specialis* applying to peace agreements and/or post-conflict situations.^109^ A further objection is that characterising an instrument as a peace agreement, and for that reason applying different rules for its interpretation, presupposes what must itself be the result of interpretation.^110^ There are also reasons to doubt such a position as a matter of policy. Peace agreements incorporate binding international legal obligations in order to guarantee that commitments made in the agreement will be delivered.^111^ The very familiarity and predictability of the established rules of treaty law, including on interpretation, are surely central to treaty obligations within peace agreements being able to fulfil this function, which would be undermined if peace agreements were interpreted using novel, specialised rules which may produce unforeseen outcomes.

The fact that a treaty is or forms part of a peace agreement will not be irrelevant to its interpretation. The rules of treaty interpretation codified in article 31 VCLT are sufficiently flexible to allow an interpreter to take account of a treaty’s object and purpose as a peace agreement within those rules. For example, besides human rights treaties, it is widely accepted that while it is the general rules of treaty interpretation that are applied to the interpretation of constituent treaties of international organisations, the flexibility of those rules allows an interpreter to take account of the treaty’s character as a constituent instrument.^112^ The different elements of interpretation will be given greater or lesser emphasis than if it were a multilateral treaty of a purely conventional character,^113^ and in particular, in the interpretation of constituent instruments, emphasis is placed on the object and purpose of the treaty.^114^ However, this is still accommodated within the existing rules of treaty interpretation and is subject to their limits. Article 31(1) requires that all the elements set out in that provision be taken into account in arriving at an interpretation, and placing greater emphasis on one provision cannot justify disregarding any of the others. Thus, if the MPA were a treaty subject to interpretation as such, its nature as a peace agreement could be taken into account and may justify greater emphasis being placed on its object and purpose in its interpretation. However, even if this were the case, this would still not be a licence to engage in a particularly generous interpretation, or one disregarding its text.

### B. Rejecting a ‘Strict Textual’ Approach

How, then, should the MPA be interpreted in article 2(1) cases? What principles should the courts apply when interpreting the rights in the RSEO part of the 1998 Agreement? In *Dillon*, at least in the High Court, the question of how the MPA should be interpreted appears to have been argued, and analysed, as a binary choice between as a treaty (generous and purposive interpretation under the VCLT rules) and as ‘not-a-treaty’. It has been argued above that the MPA is not a treaty; the position also taken by the UK government in *Dillon*. If it is not a treaty, then what exactly is the MPA, and what principles apply to its interpretation? It is argued here that the MPA is best understood as a *sui generis* constitutional document which, in accordance with both international law and existing UK constitutional law precedents, should be given a generous and purposive interpretation.

If the MPA is not a treaty, then it is not clear that international law imposes any particular rules for its interpretation. All that the rules of treaty interpretation require is that, in interpreting article 2(1), the ordinary meaning of the terms that constitute the *renvoi* (‘rights, safeguards or equality of opportunity, as set out in that part of the 1998 Agreement entitled Rights, Safeguards and Equality of Opportunity’) are determined by reference to the external norm: the MPA. As discussed above, the rules of treaty interpretation do not prescribe how that external norm is itself to be interpreted. In applying and interpreting article 2(1), the UK courts should—from the perspective of international law—adopt whatever interpretative approach to the MPA is most appropriate to its nature.

The UK government in *Dillon* argued that a ‘strict’ approach was the correct way to interpret the MPA.^115^ However, this is not sustainable. First, even if the MPA were to be interpreted as a domestic statute, the statutory interpretation rules applied by UK courts do not require ‘strict textual’ interpretation. As recently stated by Professor (now Lord) Burrows: although ‘it is not easy to pin down the present approach of the courts … it is tolerably clear today that our judges have moved from an old literal to a modern contextual and purposive approach’.^116^ Indeed, it is doubtful whether ‘strict textual’ interpretation was ever an approach adopted by UK courts in construing statutes.^117^ While the differences in approach to statutory interpretation between common law and civil law countries may be overstated, the approaches of both UK and international law can thus be contrasted with the interpretation of written law by French courts, where primacy is given to a literal interpretation.^118^ Under ordinary UK rules of statutory interpretation, ‘legislation is construed according to the ordinary meaning of the words used’ and ‘it is proper to have regard to the purpose if help is needed as to what the words actually mean’.^119^ Statutory interpretation is also ‘an exercise which requires the court to identify the meaning borne by the words in question in the particular context’.^120^

Thus, even if the MPA were a UK statute, the interpretative rules to be applied do not appear to differ significantly from the general rule in article 31(1) VCLT, which provides that a treaty shall be interpreted ‘in good faith in accordance with the ordinary meaning to be given to the terms of the treaty in their context and in the light of its object and purpose’. One could perhaps draw a distinction between the VCLT rule, which *requires* that the object and purpose of a treaty be taken into account in interpreting a treaty provision, and statutory interpretation, which merely permits the interpreter to consider the purpose where the meaning of the words is unclear. However, given the lack of clarity in how the words in the RSEO section of the MPA should be understood, this test would clearly be met in article 2(1) cases, with the result that the purpose could be considered, even under rules of statutory interpretation.

Indeed, arguably no special justification or particular legal basis is ever required for a court to consider a document’s purpose in interpreting its text. Whether an instrument is a treaty, a statute or *sui generis*, consideration of a text’s purpose is always available as a part of the interpretative process.^121^ Lord Hoffmann, writing extrajudicially, has cited article 31(1) VCLT as a succinct encapsulation of ‘the usual approach to legal instruments’ generally.^122^ It is rather the departure from this approach, and the application of a ‘strict textual’ approach which severely minimises or ignores the purpose of the instrument, that calls for justification.

In the article 2(1) cases to date, more recent decisions of the NI courts appear to have followed this approach. The Court of Appeal in *Dillon*, having spent some time setting out the parties’ arguments on treaty interpretation and concluding that ‘the VCLT applies to the interpretation exercise to be carried out by the court in relation to the RSE section of the B-GFA’ for the reasons given by the High Court judge, ultimately does not rely on these findings to decide the question of how it should be interpreted and appears to reserve its position as to whether the VCLT interpretative approach applies at all.^123^ The Court of Appeal rejects the argument that the MPA should be interpreted ‘restrictively’^124^ and takes a purposive approach in its interpretation of the MPA, apparently not on the basis that it is subject to interpretation under the VCLT rules, but because ‘There is *no reason*, in our view, to construe the broad language of the RSE chapter restrictively’.^125^

Delivered just three months after the High Court decision in *Dillon*, in *In re the Illegal Migration Act 2023* Mr Justice Humphreys takes an approach closer to that of the later Court of Appeal judgment. The judge notes that the MPA ‘was the product of lengthy negotiations between political parties, the UK and Irish Governments, and contains statements of aspiration as well as legal right’.^126^ However, the judge does not characterise the MPA as a treaty. Concluding that the rights of asylum seekers come within ‘civil rights’, Mr Justice Humphreys states that:

Reading the B-GFA as a whole, it is apparent that its provisions and protections were broad in scope … In arriving at this conclusion, I have not found it necessary to engage with the ‘generous and purposive approach’ advocated by the Vienna Convention on the Law of Treaties but have relied on a first principles approach to the interpretation of the document.^127^

The judge’s reasoning thus seems to suggest that the language of the MPA is sufficiently broad that a particularly ‘generous’ interpretation was not required: ‘The starting point of the analysis is that the RSE provisions contain a specific commitment to the “civil rights … of everyone in the community”, which must extend to asylum seekers as well as UK or Irish citizens.’^128^ The general rules of legal interpretation, which permit recourse to its purpose as a matter of course, were more than sufficient.

### C. Interpreting Constitutional Documents under UK Law

Given the broad language of the MPA, the judges in the Court of Appeal in *Dillon* and the High Court in *In re the Illegal Migration Act* were easily able to conclude that the rights of both victims and asylum seekers respectively came within the protection of article 2(1) without needing to rely on a particularly ‘generous and purposive’ interpretative approach.^129^ However, as further cases are brought which test the limits of the no diminution obligation, whether the courts of NI have the ability, or the obligation, to take not only a purposive but a ‘generous’ approach to interpreting the MPA may have significant consequences for the scope of rights protection under the I/NI Protocol. In addition to those rights specifically affirmed in the RSEO section—such as the rights to free political thought and freedom and expression of religion—can UK Acts of Parliament be disapplied in NI where they result in a diminution of other rights, such as the right to privacy, property, education or a clean environment? A ‘generous’ interpretation could make all the difference.

There are multiple reasons why applying the VCLT rules to the MPA is not a convincing justification for a ‘generous’ interpretative approach. However, ‘presumptions favouring broad, and purposive, interpretations’ are common to *constitutional* interpretation across jurisdictions.^130^ UK courts have long recognised that constitutional instruments ‘call for a generous interpretation avoiding … “the austerity of tabulated legalism”’.^131^ In *Ministry of Home Affairs v Fisher*, the House of Lords was called on to interpret the Constitution of Bermuda, an instrument scheduled to an English statutory instrument which was itself made under an Act of the UK Parliament. As analysed in the sole judgment of Lord Wilberforce, the instrument in question was ‘a Constitution, brought into force certainly by Act of Parliament, the Bermuda Constitution Act 1967 United Kingdom, but established by a self-contained document’. Thus, like the MPA, the interpretative approach to be applied to this instrument was not immediately clear. Also like the MPA, the Constitution is ‘drafted in a broad and ample style which lays down principles of width and generality’, and sets down individual rights guarantees.^132^

In response to the question of ‘whether the appellants’ premise, fundamental to their argument, that these provisions are to be construed in the manner and according to the rules which apply to Acts of Parliament, is sound’, Lord Wilberforce took the ‘more radical’ approach, which ‘would be to treat a constitutional instrument such as this as *sui generis*, calling for principles of interpretation of its own, suitable to its character as already described’.^133^ This does not mean that no rules apply to the interpretation of such an instrument,^134^ but rather

to take as a point of departure for the process of interpretation a recognition of the character and origin of the instrument, and to be guided by the principle of giving full recognition and effect to those fundamental rights and freedoms with a statement of which the Constitution commences.^135^

In the more recent case of *Reye*s, another post-colonial constitutional interpretation case, the Privy Council was called to interpret the Constitution of Belize. In the single judgment, Lord Bingham set out his approach to constitutional interpretation:

As in the case of any other instrument, the court must begin its task of constitutional interpretation by carefully considering the language used in the Constitution. But it does not treat the language of the Constitution as if it were found in a will or a deed or a charterparty. A *generous and purposive* interpretation is to be given to constitutional provisions protecting human rights.^136^

Although the post-colonial context of *Fisher* and *Reyes* is clearly different from that of the 1998 Agreement, the question of interpretation raised is strikingly similar and it is submitted that the approach of the House of Lords and Privy Council in interpreting the rights provisions in those cases is also the most appropriate approach to interpreting the MPA in article 2(1) cases. The MPA is an annex to a treaty which has been partly incorporated into UK law through the Northern Ireland Act and is referred to by the WA, another treaty which has been partly incorporated into domestic law. That is, like the Constitution of Bermuda, it is a *sui generis* instrument connected to other legal instruments to which clear rules of interpretation apply—a statute; a treaty—but whose own nature and means of interpretation do not fit clearly into existing legal categories. As Campbell, Ní Aoláin and Harvey have argued, ‘the norms of international law alone will not explain or contain all the elements which constitute the Good Friday Agreement … the document is best understood as a hybrid, containing elements of constitutional settlement and international legal discourse’.^137^

Moreover, although not explicitly labelled as such, the MPA clearly, in relation to NI, possesses features that distinguish constitutions. In Strand One, it defines the constitution and powers of the main organs of the different branches of government; although not explicitly stated, from the content of the agreement, which makes provision for ‘the future’, it is meant to be of long duration, and has already endured more than 25 years; and it has a canonical formulation, enshrined in the annex to the BIA. Moreover, especially in light of its endorsement in referenda on either side of the border, ‘its provisions include principles of government … that are generally held to express the common beliefs of the population about the way their society should be governed’.^138^ Finally, like the Constitution of Bermuda, the MPA contains a statement of rights and freedoms: the RSEO part of the agreement whose interpretation is at issue in article 2(1) cases. Indeed, the only constitutional features which the MPA seems to lack are those on which the UK constitution generally falls down: that it constitutes a superior law which invalidates conflicting ordinary law; and that there are judicial procedures to implement the superiority of the constitution.^139^ The MPA is thus best understood as ‘a new constitutive act with normative status which confronts traditional UK constitutional law directly’.^140^

In the context of NI specifically, support for a ‘generous and purposive’ approach to interpretation of the MPA *qua* constitutional instrument may already be found in a more modern precedent which has been neglected in recent years, but which in light of the article 2(1) cases has acquired a new relevance. The phrase ‘generous and purposive’ is found in another celebrated judgment by Lord Bingham, three years prior to *Roma Rights* but, more significantly, a mere six months subsequent to his judgment applying a ‘generous and purposive’ interpretation to the Constitution of Belize in *Reyes*. In *Robinson v Secretary of State for Northern Ireland*, which dealt with the interpretation of the 1998 Northern Ireland Act, Lord Bingham stated that:

The 1998 Act does not set out all the constitutional provisions applicable to Northern Ireland, but it is in effect a constitution. So to categorise the Act is not to relieve the courts of their duty to interpret the constitutional provisions in issue. But the provisions should, consistently with the language used, be interpreted generously and purposively, bearing in mind the values which the constitutional provisions are intended to embody.^141^

It is unclear whether, when setting out his approach to interpretation of a treaty with humanitarian purposes three years later in *Roma Rights*, Lord Bingham chose to echo the language he had used to characterise his constitutional approach to interpretation of the 1998 Act, which was decisive in *Robinson*. There is no explicit reference to the earlier judgment, but this is not unexpected since, although there is a superficial similarity between the two cases—both addressed the question of how to interpret an Act of Parliament that has been adopted to implement another instrument, the 1951 Convention and the 1998 Agreement respectively—in *Robinson* there was no reference to the VCLT and no suggestion that the MPA was a treaty. Indeed, the judges in *Robinson* are silent as to what exactly they think the MPA is, and there is nothing to suggest they think it has any binding force in international or domestic law. Lord Hoffmann includes it within the category of ‘facts and documents [that] form part of the admissible background for the construction of the Act just as much as the Revolution, the Convention and the Federalist Papers are the background to construing the Constitution of the United States’.^142^

This constitutional approach in *Robinson* provides a more plausible alternative basis for a ‘generous and purposive’ interpretation of the RSEO chapter of the MPA in article 2(1) cases. It is true that, in the two decades since *Robinson*, the UK’s highest court has generally favoured an approach to the interpretation of constitutional statutes which is

arguably defined by an unwavering reliance on, and attentiveness to, the language of the relevant statutory provisions alone, with little contemplation for how, as part of the interpretative process, that language should be reconciled with, or understood alongside, other components of the constitution.^143^

In particular, the Supreme Court has been reluctant to engage with the full constitutional and political significance of the Northern Ireland Act.^144^ As a matter of policy, however, a constitutional vision that ‘overlooks whether a court’s interpretative approach is or ought to be shaped by the nature and extent of the constitutional character of the provisions or norms’^145^ both is inadequate to address the far-reaching consequences of Brexit for the UK constitution, many of which remain to be fully worked out, and risks sidelining the political as well as legal nature of NI’s ‘unique constitutional settlement’.^146^

Even if the approach of the Supreme Court to interpret devolution statutes in the same way as any other statute is correct in the Welsh and Scottish contexts, when it comes to NI a ‘problem arises when legal technique is unable to capture the complexity of the political context’ as ‘dominant constitutional law doctrine is constructed on the assumption of normality and the absence of violence as a defining feature of the state’s existence’.^147^ Twenty-five years after the 1998 Agreement was signed, it would still be both naive and complacent to assume that the NI devolution settlement can be interpreted in the same manner as that of the other nations. And even if the lower courts felt unable to invoke the interpretative approach set out in *Robinson*, given subsequent precedents taking a more conservative approach, no such constraint applies to the apex court.

More significantly, even if there are precedents demonstrating that the highest court’s approach to *statutory* interpretation in constitutional cases has become more conservative since *Robinson*,^148^ the issue in the article 2(1) cases is not the proper approach to interpretation of the Northern Ireland Act or 2018 Act or any other statute, but of the MPA itself, and later constitutional statute cases may thus be distinguished. In *Robinson*, the House of Lords took a ‘generous and purposive’ approach to interpretation not of the MPA, but of the 1998 Act which sought to implement it. However, it was the nature of the MPA it incorporated as a political and constitutional document that led Lord Bingham and the majority to conclude that the 1998 Act was, in effect, a constitution for NI. Thus, if the Act should be given a ‘generous and purposive’ interpretation on this basis, surely the same goes *a fortiori* for the MPA itself. In this way, it is *Fisher* and the post-colonial constitution cases that provide the closer precedent for the interpretation of the MPA. Those cases deal not with interpretation of *a statute implementing* a constitution or other instrument, but with the interpretation of the constitution itself. Unlike *Robinson*, which dealt with interpretation of the 1998 Act *qua* statute implementing the 1998 Agreement, in article 2(1) cases the *renvoi* to the RSEO section means it is the interpretation of the MPA itself that is at issue. For this reason, despite the very different contexts, this similarity in the structure of the question asked and the nature of the instrument being interpreted bring article 2(1) cases within the approach in *Fisher* and *Reyes*: constitutions must be given a ‘generous and purposive’ interpretation, particularly when they contain provisions protecting fundamental human rights.

## 5. Conclusion

Of the article 2(1) I/NI Protocol cases to date, only the High Court and, to a lesser extent, the Court of Appeal in *Dillon* go into detail in their analysis of how the ‘part of the 1998 Agreement entitled Rights, Safeguards and Equality of Opportunity’ should be interpreted. Although this attention to the question of interpretation of the 1998 Agreement is welcome, both judgments demonstrate some laxity in how key international law principles and concepts—in particular, the scope of application of relevant treaties, and the relationship between treaty and custom—are applied, and the conclusion that the MPA should be interpreted as an international treaty subject to the VCLT rules is difficult to sustain.

As the NI courts face future article 2(1) cases, the better approach is, first of all, to reject the false choice between a ‘generous and purposive’ interpretation that is available only through application of the VCLT and a ‘strict textual’ approach. This was the approach correctly taken by the Court of Appeal in *Dillon* and the High Court in *In re the Illegal Migration Act 2023*, whose decisions recognised implicitly that regard to the purpose of an instrument does not require application of the VCLT rules and it is instead a strict interpretation that calls for justification. Looking further ahead, the full implications of the obligation created by article 2(1), and in particular the scope of rights protected through the RSEO part of the MPA, remain to be explored. As more cases come before the courts, the question of whether a ‘generous and purposive’ approach to interpretation can be taken may therefore become more significant in determining the limits of the article 2(1) obligation.

The MPA should be given a generous and purposive approach, but not on the erroneous basis that it is a treaty which should be interpreted using the VCLT rules. The UK courts have existing domestic precedent which supports the application of a ‘generous and purposive’ approach when interpreting the MPA, both under UK law and consistent with international treaty law: the House of Lords’ 2002 judgment in *Robinson*, alongside precedents on the interpretation of constitutions of former British colonies. Indeed, as Brexit brings NI issues increasingly before the UK courts, it is disappointing that they have not, as yet, demonstrated the constitutional vision and facility with international law that was so characteristic of Lord Bingham’s judgments. As the first article 2(1) case proceeds before the Supreme Court, the Court has an opportunity to clarify the reasoning of the earlier article 2(1) decisions in relation to the issues of international law they raise, but also to recognise the MPA’s true nature as, in effect, NI’s ‘foundational constitutional document’,^149^ which should be interpreted as such.

## Footnotes

1 Agreement on the withdrawal of the United Kingdom of Great Britain and Northern Ireland from the European Union and the European Atomic Energy Community, Protocol on Ireland/Northern Ireland (signed 24 January 2020, entered into force 1 February 2020, with all its provisions applying since 1 January 2021).

2 Decision 1/2023 of the Withdrawal Agreement Joint Committee on laying down arrangements relating to the Windsor Framework (24 March 2023) para 4. This article will continue to refer to the Windsor Framework as the ‘I/NI Protocol’, as this more clearly reflects that instrument’s nature as a protocol to an international treaty.

3 Anurag Deb and CRG Murray, ‘Article 2 of the Windsor Framework, the Legacy Act and the Illegal Migration Act: Adjusting to Post-Brexit Realities’ [2025] PL 477, 480; see UK government commitment to ‘no diminution of rights, safeguards and equality of opportunity’ in Northern Ireland (2020) <https://assets.publishing.service.gov.uk/media/5f2d14f7d3bf7f1b10d58f8c/Explainer__UK_Government_commitment_to_no_diminution_of_rights__safeguards_and_equality_of_opportunity_in_Northern_Ireland.pdf> paras 1 and 4–5.

4 Art 4(1) continues: ‘Accordingly, legal or natural persons shall in particular be able to rely directly on the provisions contained or referred to in this Agreement which meet the conditions for direct effect under Union law.’ Similarly, s 7A of the 2018 Act protects rights and obligations ‘as in accordance with the withdrawal agreement are without further enactment to be given legal effect or used in the United Kingdom’. The Court of Appeal in *Dillon* concluded that art 2(1) is directly effective, *Dillon and others’ applications for judicial review* [2024] NICA 59 [85] (*Dillon Appeal*). This has been challenged by the UK government before the Supreme Court but is not relevant to the arguments in this article.

5 Inserted by s 5 of the European Union (Withdrawal Agreement) Act 2020. See *In re Allister’s and Peeples’ applications for judicial review* [2023] UKSC 5 [73]–[74] (Lord Stephens).

6
*R v Secretary of State for Transport, ex parte Factortame Ltd (No 2)* [1991] 1 AC 603. See *In re Illegal Migration Act* [2024] NIKB 35 [53] (*IMA*).

7 European Union (Withdrawal) Act 2018, s 7A(3).

8
*Dillon and others’ applications for judicial review* [2024] NIKB 11 (*Dillon*).

9 The judgment also made significant findings as to the incompatibility of the Act with the ECHR. See Anurag Deb and Colin Murray, ‘Dillon and Others’ Applications for Judicial Review—A Radically Unradical Analysis of the Legacy Act’ (18 March 2024) <https://ukhumanrightsblog.com/2024/03/18/dillon-and-others-applications-for-judicial-review-a-radically-unradical-analysis-of-the-legacy-act/>.

10
*Dillon* (n 8) [527].

11 See *In re SPUC* [2023] NICA 35 [54]; *In re Disability Action* [2025] NIKB 39 [64]–[75].

12 Deb and Murray (n 3) 478.

13 See <www.supremecourt.uk/cases/uksc-2025-0013>. Although the new Labour government has introduced legislation to repeal and replace the Act which is the subject of the *Dillon* litigation (see <www.gov.uk/government/news/northern-ireland-troubles-bill-to-repeal-and-replace-legacy-act>), the government is appealing the Court of Appeal judgment.

14 See <https://goodlawproject.org/wp-content/uploads/2025/07/REDACTED-2025.07.11-Good-Law-Project-Another-Application-for-Leave-to-Apply-for-Judicial-Review.pdf>.

15 NIHRC Briefing on the Data Protection and Digital Information Bill (29 July 2025) <https://nihrc.org/publication/detail/nihrc-briefing-on-the-data-protection-and-digital-information-bill>.

16 International Law Commission, ‘Articles on the Responsibility of States for Internationally Wrongful Acts with Commentaries’ in ‘Report of the International Law Commission, 53rd session’ (23 April–1 June and 2 July–10 August 2001) UN Doc A/56/10, 43, art 3.

17 EU–UK Withdrawal Agreement, Title III, art 170.

18 The WA provides only that ‘provisions of this Agreement referring *to Union law or to concepts or provisions thereof* shall be interpreted and applied in accordance with the methods and general principles of Union law’ (Art 4(3)) (emphasis added) and that questions of interpretation of a concept or provision of Union law will be referred by the arbitration panel to the CJEU for a ruling (Art 174(1)). See *Dillon Appeal* (n 4) [81].

19 Philip Sales and Joanne Clement, ‘International Law in Domestic Courts: The Developing Framework’ (2008) 124 LQR 388, 401 fn 63.

20 See I/NI Protocol, arts 2(2), 11(1) and 18(2) and (4).

21 Anurag Deb and CRG Murray ‘Article 2 of the Ireland/Northern Ireland Protocol: A New Frontier in Human Rights Law?’ (2023) 6 EHRLR 608, 611; Christopher McCrudden, ‘The Origins of “Civil Rights and Religious Liberties” in the B-GFA’ (2024) 75(1) NILQ 29, 34.

22 Agreement between the Government of the United Kingdom of Great Britain and Northern Ireland and the Government of Ireland (with annexes) (adopted 10 April 1998, entered into force 2 December 1999) 2114 UNTS 473.

23 See, eg House of Commons Northern Ireland Affairs Committee, ‘The Effectiveness of the Institutions of the Belfast/Good Friday Agreement: First Report of Session 2023–24’ (29 November 2023) <https://committees.parliament.uk/publications/42405/documents/210752/default/>; Conor J Kelly, Alan Renwick and Alan Whysall, ‘Reform of Stormont: Options for Discussion’ (UCL Constitution Unit, March 2025).

24 See Christopher McCrudden, Oran Doyle and David Kenny, ‘The Franchise in Irish Unification Referendums’ (2021) 32(2) Irish Studies in International Affairs 183; Oran Doyle and others, ‘Legal Convergence and Divergence on the Island of Ireland: Report of the North–South Legal Mapping Project to the Shared Island Unit’ (Trinity College Dublin, September 2022).

25 Colin J Harvey, ‘The New Beginning: Reconstructing Constitutional Law and Democracy in Northern Ireland’ in Colin J Harvey (ed), *Human Rights, Equality and Democratic Renewal in Northern Ireland* (OUP 2001) 9, 36.

26
*In re Caoimhe Ní Chuinneagain’s application for judicial review* [2021] NIQB 79 [20]–[28] (Scoffield J).

27 [2022] NICA 56 [8]–[9], [15], [62]–[66] and [108] (McCloskey LJ).

28
*In re SPUC* [2022] NIQB 9 [145] (Colton J) (emphasis added).

29 [2023] NICA 35 [56] (Keegan LCJ).

30
*IMA* (n 6) [70]. This decision is discussed further in section 4 below.

31
*Dillon* (n 8) [63] and [532].

32 ibid [542]–[549].

33 ibid [529], [561], [570]–[573] and [578].

34
*Dillon Appeal* (n 4).

35
*Dillon* (n 8) [533].

36 Brice Dickson, ‘The Meaning of “Civil Rights” in the Belfast (Good Friday) Agreement’ (*UK Constitutional Law Blog*, 13 October 2025) <https://ukconstitutionallaw.org/2025/10/13/brice-dickson-the-meaning-of-civil-rights-in-the-belfast-good-friday-agreement/>.

37 Colm Campbell, Fionnuala Ní Aoláin and Colin Harvey, ‘The Frontiers of Legal Analysis: Reframing the Transition in Northern Ireland’ (2003) 66(3) MLR 317, 321–3.

38 Vienna Convention on the Law of Treaties (adopted 23 May 1969, entered into force 27 January 1980) 1155 UNTS 332, art 2.

39 1986 Vienna Convention on the Law of Treaties between States and International Organizations or between International Organizations (signed 21 March 1986).

40 VCLT, art 4.

41 Mark Villiger, *Commentary on the 1969 Vienna Convention on the Law of Treaties* (Nijhoff 2009) 111.

42 It is beyond doubt that the general rule of interpretation in art 31(1) VCLT and VCLT-IO also exists as a rule of customary international law, International Court of Justice, *Legality of Use of Force (Serbia and Montenegro v Belgium)* (Preliminary Objections) [2004] ICJ Reports 279, para 100.

43 International Court of Justice, *Gabcikovo–Nagymaros Project* (Hungary/Slovakia) (Judgment) [1997] ICJ Reports 7, para 109. See Villiger (n 41) 59.

44 See Sales and Clement (n 19) 413–14; *R v Jones* [2006] UKHL 16 (Lord Bingham) [11]. cf *Trendtex Trading Corp v Central Bank of Nigeria* [1977] QB 529, 554G (Lord Denning); *Campaign for Nuclear Disarmament v Prime Minister* [2002] EWHC 2777 (Admin) [23].

45
*Belhaj v Straw* [2017] UKSC 3 [252] (Lord Sumption).

46 ibid. See also ‘where the constitution permits’ *R v Jones* (n 44) [23].

47
*JH Rayner (Mincing Lane) Ltd v International Tin Council* [1990] 2 AC 418 [476H]–[477A]; *EOG v Home Secretary* [2022] EWCA Civ 307 [35]. But cf *R (SG) v Secretary of State for Work and Pensions* [2015] UKSC 16 [235]–[257] (Lord Kerr).

48
*R v Lyons* [2003] 1 AC 976 [27] (Lord Hoffmann).

49 Sales and Clement (n 19) 403; *A v Home Secretary (No 2)* [2005] 1 WLR 414 [266]–[267] (Laws LJ).

50
*Dillon Appeal* (n 4) [75]; see also [105].

51 ibid [78].

52 Christine Bell, *On the Law of Peace: Peace Agreements and the Lex Pacificatoria* (OUP 2008) 128–35 and 142–3.

53 ibid 161.

54 Mark Retter, Andrea Varga and Marc Weller, ‘Introduction: Framing the Relationship between International Law and Peace Settlements’ in Mark Retter and others (eds), *International Law and Peace Settlements* (CUP 2021) 1, 16. See also Christine Bell, ‘Peace Agreements: Their Nature and Legal Status’ (2006) 100(2) AJIL 373, 379.

55 This definition of ‘treaty’ is accepted to reflect customary international law, see Villiger (n 41) 83. See also International Court of Justice, *Maritime Delimitation and Territorial Questions Between Qatar and Bahrain* (Jurisdiction and Admissibility) Judgment [1994] ICJ Reports 112, para 23.

56
*Dillon* (n 8) [533].

57
*Dillon Appeal* (n 4) [79].

58
*Dillon* (n 8) [533].

59 BIA, Preamble, recital 1.

60 See Villiger (n 41) 104.

61 VCLT, art 3.

62 Robert E Dalton, ‘International Documents of a Non-Legally Binding Character’ reproduced in Marian Nash (Leich), ‘Contemporary Practice of the United States Relating to International Law’ (1994) 88 AJIL 515.

63
*Dillon* (n 8) [533].

64 Harvey shares the view that, as an annex, the MPA is ‘an intrinsic part of a bilateral and binding international legal agreement’, Colin Harvey, ‘The 1998 Agreement: Context and Status’ in Chris McCrudden (ed), *The Law and Practice of the Northern Ireland Protocol* (OUP 2021) 21 and 26. cf Asli Ozcelik, ‘Entrenching Peace in Law: Do Peace Agreements Possess International Legal Status?’ (2020) 21 MJIL 1, 11 fn 37.

65 Jeremy Hill, *Aust’s Modern Treaty Law and Practice* (4th edn, CUP 2023) 432.

66 See, eg Convention on International Trade in Endangered Species of Wild Fauna and Flora (signed 3 March 1973, entered into force 1 July 1975) 993 UNTS 243, arts XV–XVII.

67 Villiger (n 41) 81.

68
*Dillon* (n 8) [534].

69 See Villiger (n 41) 30.

70 International Court of Justice, *Aegean Sea Continental Shelf (Greece v Turkey)*, (Jurisdiction) [1978] ICJ Reports 3, para 107.

71 BIA, art 2; *Dillon* (n 8) [534]. See also Maria Xiouri, ‘The reform of the institutions created by the 1998 Belfast/Good Friday Agreement and treaty amendment: an example of the interplay between a treaty and a non-legally binding agreement’ (2025) 76(3) NILQ 530, 538–9.

72 Bell, ‘Peace Agreements’ (n 54) 399.

73 McCrudden, ‘The Origins of “Civil Rights and Religious Liberties”’ (n 21) 66.

74
*Dillon* (n 8) [534].

75 Emphasis added.

76
*Dillon Appeal* (n 4) [87].

77 Duncan French, ‘Treaty Interpretation and Extraneous Legal Rules’ (2005) 55 ICLQ 281, 294.

78 See Catherine Redgwell, ‘The Never-Ending Story: The Role of GAIRS in UNCLOS Implementation’ in Jill Barrett and Richard Barnes (eds), *Law of the Sea: UNCLOS as a Living Treaty* (BIICL 2016) 183.

79 Dapo Akande and Katie A Johnston, ‘Implications of the Diversity of the Rules on the Use of Force for Change in the Law’ (2021) 32 EJIL 679, 687–8.

80 Emphasis added.

81 The Court does not seem persuaded by this argument, taking the view that while EU law interpretive principles may apply to parts of the WA, ‘the full WA itself is not treated in that way’: *Dillon Appeal* (n 4) [80]–[81]. See also *Secretary of State for Work and Pensions v AT* [2023] EWCA Civ 1307 [80] (Green LJ).

82 As perhaps suggested in *Dillon Appeal* (n 4) [43].

83 Bell, *On the Law of Peace* (n 52) 146–7.

84 International Law Commission, UN Doc A/56/10 (n 16) 43, arts 1–2. These provisions reflect customary international law.

85 See eg VCLT, art 54 (Termination or withdrawal through consent), art 60 (Termination or suspension as a consequence of breach) and arts 39–40 (Amendment).

86 See Bell, ‘Peace Agreements’ (n 54) 399.

87 Full argument is beyond the scope of this article, but see Katie A Johnston, ‘Unilateral Change to the Institutions of the 1998 Belfast/Good Friday Agreement’ (2025) 76(3) NILQ 554.

88 Particularly given recent comments by certain political actors. See eg Sam Francis, ‘UK Will Leave ECHR if Tories Win Election, Badenoch Says’ (*BBC News*, 3 October 2025) <www.bbc.co.uk/news/articles/c1mxy2j2elro>; Andrew Madden, ‘Nigel Farage: Reform UK Would Renegotiate Good Friday Agreement as Part of Immigration Plan’ *Belfast Telegraph* (26 August 2025) <https://m.belfasttelegraph.co.uk/news/politics/nigel-farage-reform-uk-would-renegotiate-good-friday-agreement-as-part-of-immigration-plan/a2034346170.html>.

89 [2005] 2 AC 1 [18].

90 Richard Gardiner, *Treaty Interpretation* (2nd edn, OUP 2015) 9 and 464–5.

91 Georg Nolte, ‘Still Setting the Frame: The Vienna Convention’s Rules on Treaty Interpretation’ (2021) 24(1) Austrian Review of International and European Law 53, 57.

92 International Law Commission, ‘Draft Articles on the Law of Treaties with Commentaries’ (1966) II United Nations Yearbook of the International Law Commission 187, 219–20 para 8.

93 See Gardiner (n 90) 211.

94 International Court of Justice, *Territorial Dispute (Libyan Arab Jamahiriya/Chad)* (Judgment) [1994] ICJ Reports 6, para 41.

95 Philipp Allott, ‘The Concept of International Law’ (1999) 10 EJIL 31, 43.

96 International Law Commission, ‘Draft Articles on the Law of Treaties’ (n 92) draft art 27, commentary paras 18–19.

97 International Court of Justice, *Dispute regarding Navigational and Related Rights (Costa Rica v Nicaragua)* (Judgment) [2009] ICJ Reports 213, para 48.

98 Ulf Linderfalk*, On the Interpretation of Treaties: The Modern International Law as Expressed in the 1969 Vienna Convention on the Law of Treaties* (Dordrecht 2007) 8; Malgosia Fitzmaurice, ‘Dynamic (Evolutive) Interpretation of Treaties’ (2008) 21 Hague Yearbook of International Law 101, 114; Villiger (n 41) 436; Gardiner (n 90) 461.

99 Villiger (n 41) 440, 428.

100 See eg Court of Justice of the European Union, *Parti écologiste ‘Les Verts’ v European Parliament*, Judgment (1986) Case 294/83 [24]–[25]; José Carlos de Carvalho Moitinho de Almeida, ‘Interprétation par la Cour de justice de l’Union du droit européen de procédure civile’ in Kieran Bradley, Noel Travers and Anthony Whelan (eds), *Of Courts and Constitutions: Liber Amicorum in Honour of Nial Fennelly* (Hart Publishing 2014) 13, 20.

101
*Dillon* (n 8) [530].

102
*Roma Rights* (n 89) [18].

103
*Secretary of State for Work and Pensions v AT* [2023] EWCA Civ 1307 [80] (Green LJ), citing *Al-Malki and another v Reyes (Secretary of State for Foreign and Commonwealth Affairs and another intervening)* [2017] UKSC 61.

104
*Roma Rights* (n 89) [18]. See *Januzi v Secretary of State for the Home Department* [2006] 2 AC 426 [4]; *R (on the application of ST (Eritrea)) (FC) v Secretary of State for the Home Department* [2012] UKSC 12 [30]. See also *Adan v Secretary of State for the Home Department* [1999] 1 AC 293 [305].

105 See Eirik Bjorge, ‘Treaty Interpretation: International Humanitarian Law and International Human Rights Law’ in Robert Kolb, Gloria Gaggioli and Pavle Kilibarda (eds), *Research Handbook on Human Rights and Humanitarian Law* (Edward Elgar 2022) 57 and 70.

106
*Al-Malki* (n 103) [11].

107 Bell, ‘Peace Agreements’ (n 54) 412.

108 Ruti G Teitel, *Transitional Justice* (OUP 2000) 4.

109 Marc Weller, ‘Conclusion’ in Retter, Varga and Weller (n 54) 685, 691.

110 Stefan Kadelbach, ‘Interpretation of the Charter’ in Simma and others (eds), *The Charter of the United Nations: A Commentary* (3rd edn, OUP 2012) paras 4 and 20 (on ‘constitutional’ interpretation of the UN Charter).

111 Bell, ‘Peace Agreements’ (n 54) 406.

112 Niels Blokker, ‘Constituent Instruments’ in Katz Cogan and others, *The Oxford Handbook of International Organizations* (OUP 2016) 954–5.

113 Dapo Akande, ‘International Organisations’ in Malcolm Evans (ed), *International Law* (5th edn, OUP 2018) 237.

114 Jan Klabbers, *An Introduction to International Organizations Law* (3rd edn, CUP 2018) 83.

115
*Dillon* (n 8) [530]–[532].

116 Andrew Burrows, *Thinking about Statutes: Interpretation, Interaction, Improvement* (CUP 2018) 5. cf Jeffrey Goldsworthy, ‘Lord Burrows on Legislative Intention, Statutory Purpose, and the “Always Speaking” Principle’ (2022) 43(1) Stat LR 79, 90.

117 Peter Benson Maxwell and Wyatt Wyatt-Paine, *On the Interpretation of Statutes* (Sweet & Maxwell 1989) 39.

118 Gerard Carney, ‘Comparative Approaches to Statutory Interpretation in Civil Law and Common Law Jurisdictions’ (2015) 36 Stat LR 46, 51–3; Joseph Dainow, ‘The Civil Law and the Common Law: Some Points of Comparison’ (1967) 15 Am J Comp L 419, 424–5.

119
*Imperial Tobacco v Lord Advocate* [2012] UKSC 61 [14]–[15] (Lord Hope); cited with approval in *Lord Advocate’s Reference (No 1) (Devolution Issues)* [2022] UKSC 31 [38] (Lord Reed). See also *R (on the application of The Project for the Registration of Children as British Citizens) v Secretary of State for the Home Department* [2022] UKSC 3 [29] (Lord Hodge).

120
*R v Secretary of State for the Environment, Transport and the Regions, ex parte Spath Holme Ltd* [2001] 2 AC 349, 396–7 (Lord Nicholls).

121 Aharon Barak and Sari Bashi, *Purposive Interpretation in Law* (Princeton UP 2005) 87; Kent Greenawalt, *Statutory and Common Law Interpretation* (OUP 2012) 97.

122 Lord Hoffmann, ‘Judges, Interpretation and Self-Government’ in Richard Ekins, Paul Yowell and NW Barber (eds), *Lord Sumption and the Limits of the Law* (Bloomsbury 2016) 67 and 68.

123
*Dillon Appeal* (n 4) [79] and [115].

124 ibid [115].

125 ibid [115] (emphasis added).

126 See *IMA* (n 6) [67].

127 ibid [70].

128 ibid [68].

129
*Dillon Appeal* (n 4) [116]–[118]; *IMA* (n 6) [67]–[68] and [70].

130 Jeffrey Goldsworthy, ‘Conclusions’ in Jeffrey Goldsworthy (ed), *Interpreting Constitutions* (OUP 2006) 321 and 325.

131
*Ministry of Home Affairs v Fisher* [1980] AC 318 [328]; see also *Matadeen v Pointu* [1999] 98 [108E] (Lord Browne-Wilkinson).

132
*Fisher* (n 131) [328].

133 ibid [329].

134 Derek O’Brien, ‘The Interpretation of Commonwealth Caribbean Constitutions: Does Text Matter?’ in Richard Albert, Derek O’Brien and Se-shauna Wheatle (eds), *The Oxford Handbook of Caribbean Constitutions* (OUP 2020) 555 and 566.

135
*Fisher* (n 131) [329].

136
*Reyes v The Queen* (PC) [2002] 2 AC 235 [26] (emphasis added).

137 Campbell, Ní Aoláin and Harvey (n 37) 333.

138 These criteria are taken from Joseph Raz, ‘On the Authority and Interpretation of Constitutions: Some Preliminaries’ in *Between Authority and Interpretation: On the Theory of Law and Practical Reason* (OUP 2009) 324.

139 Although arguably art 2(1) and the s 7A disapplication power that are the focus of this article now supply such features to some extent.

140 Harvey, ‘The New Beginning’ (n 25) 30.

141
*Robinson v Secretary of State for Northern Ireland* [2002] WL 1446153 [11]. See also *In re McComb* [2003] UKQB 47 [31] (Kerr J). On the 1998 Act, see David Feldman, ‘Statutory Interpretation and Constitutional Interpretation’ (2014) 130 LQR 473, 494.

142
*Robinson* (n 142) [33].

143 Mark Elliott and Nicholas Kilford, ‘Nothing to See Here? Allister in the Supreme Court’ (2024) 28 Edinburgh Law Review 95, 96. See eg *Re Agricultural Sector (Wales) Bill* [2014] UKSC 43 [64]–[68].

144 Notably *R (Miller) v Secretary of State for Exiting the European Union* [2017] UKSC 5 [129]–[135]; see also *Allister* (n 5) [66]. See Chris McCrudden and Daniel Halberstam, ‘Miller and Northern Ireland: A Critical Constitutional Response’ (2017) 8 UK Supreme Court Yearbook 199, 231–2 and 239; Gregory Davies, ‘The UK Supreme Court and Devolution: Guardian of the Passive Revolution?’ [2025] PL 58, 63–70.

145 Elliott and Kilford (n 143) 99.

146 McCrudden and Halberstam (n 144) 302, citing Christopher McCrudden, `Northern Ireland and the British Constitution Since the Belfast Agreement' in Jeffrey Jowell and Dawn Oliver (eds), The Changing Constitution (6th edn, OUP 2007) 227, at 268.

147 Campbell and others (n 37) 322.

148 Speaking extrajudicially in 2018, Lady Hale, then President of the Supreme Court, took the view that *Robinson* remained a relevant precedent for interpretation of devolution statutes, ‘Devolution and The Supreme Court—20 Years On’ (14 June 2018) <https://uk-supremecourt.co.uk/uploads/speech_180614_daff0d430c.pdf>.

149 Harvey, ‘The 1998 Agreement’ (n 64) 22.

